# Supplementary material for: Optogenetic frequency scrambling of hippocampal theta oscillations dissociates working memory retrieval from hippocampal spatiotemporal codes
Source: Nat Commun. 2023 Jan 25;14:410. doi: 10.1038/s41467-023-35825-5 (PMC9877037; doi:10.1038/s41467-023-35825-5)
Supplement: Supplementary file 1 — Supplementary Information [file 41467_2023_35825_MOESM1_ESM.pdf]

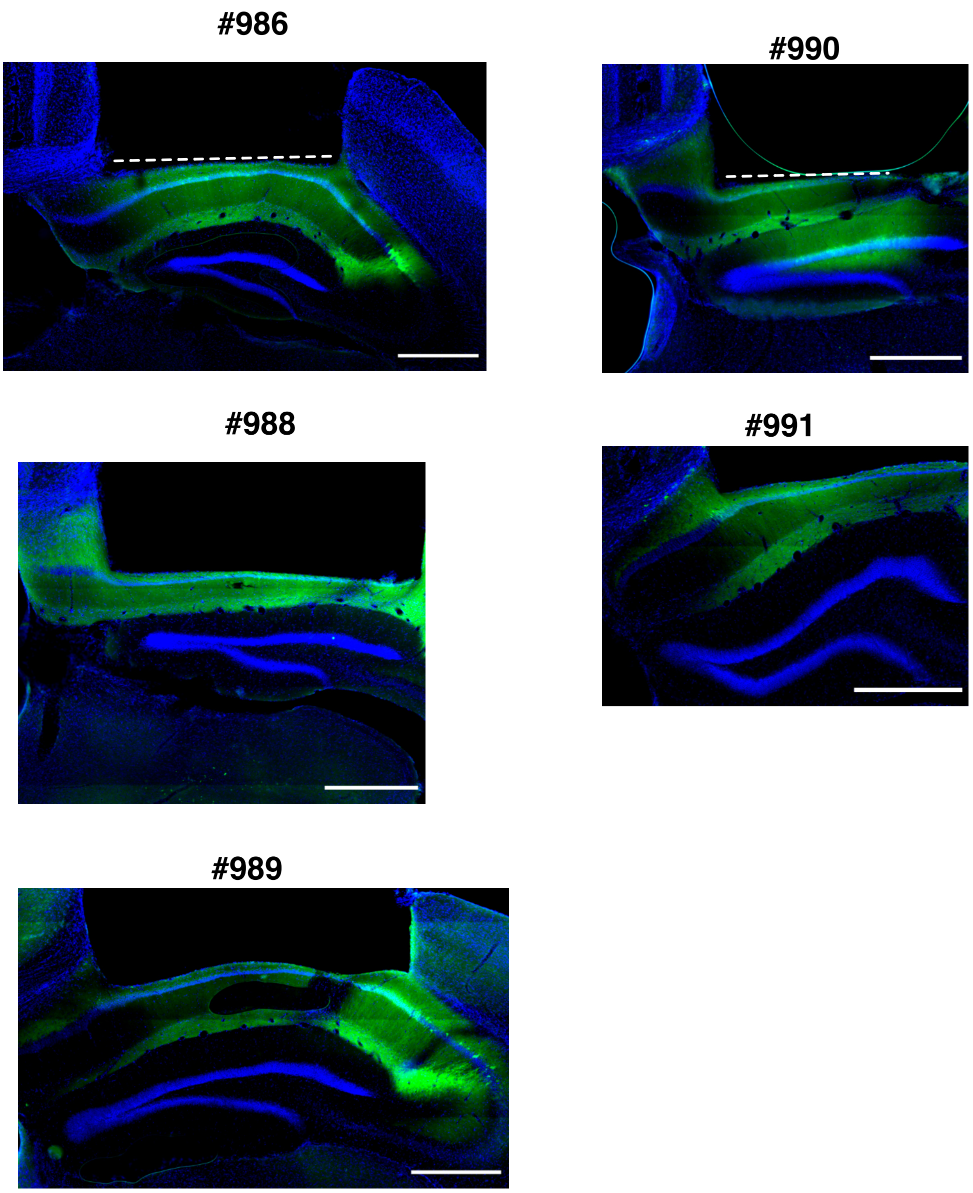

Supplementary figure 1. Histological analysis of CKII-GCamp6f transfection and lens placement. coronal sections of dorsal hippocampus displaying location of lens surface (white dotted line). blue, DAPI; green, GCaMP6f. Scale bars in all micrographs: 500 µm. All subjects are included in this study, and lens placement has been recapitulated in at least 5 independent experiments.

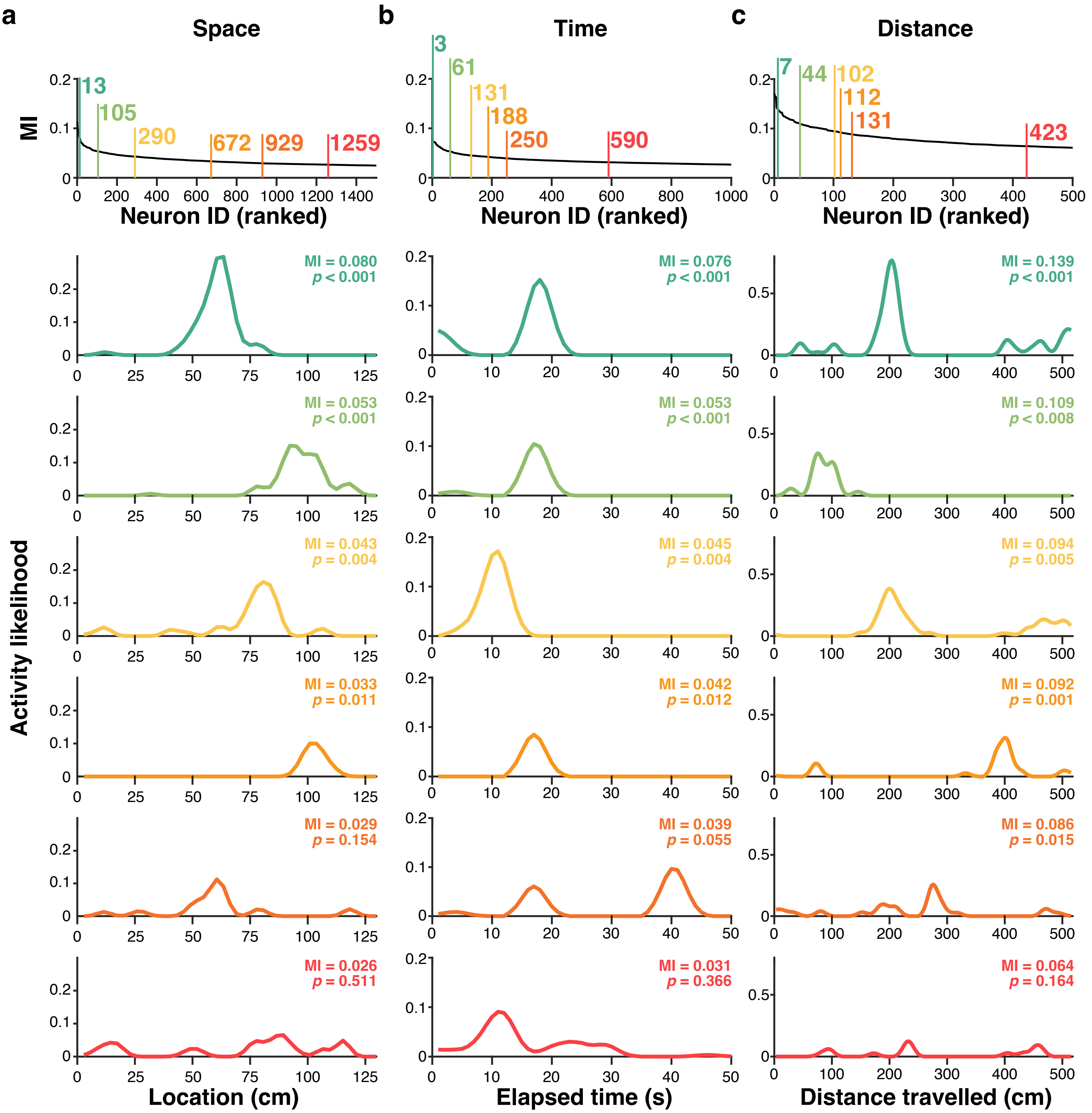

Supplementary figure 2. Example cells modulated by space (**a**), time (**b**), and distance travelled (**c**) and ranked by their mutual information. For each cell, a tuning curve with corresponding MI value and its significance (p-value) is displayed.

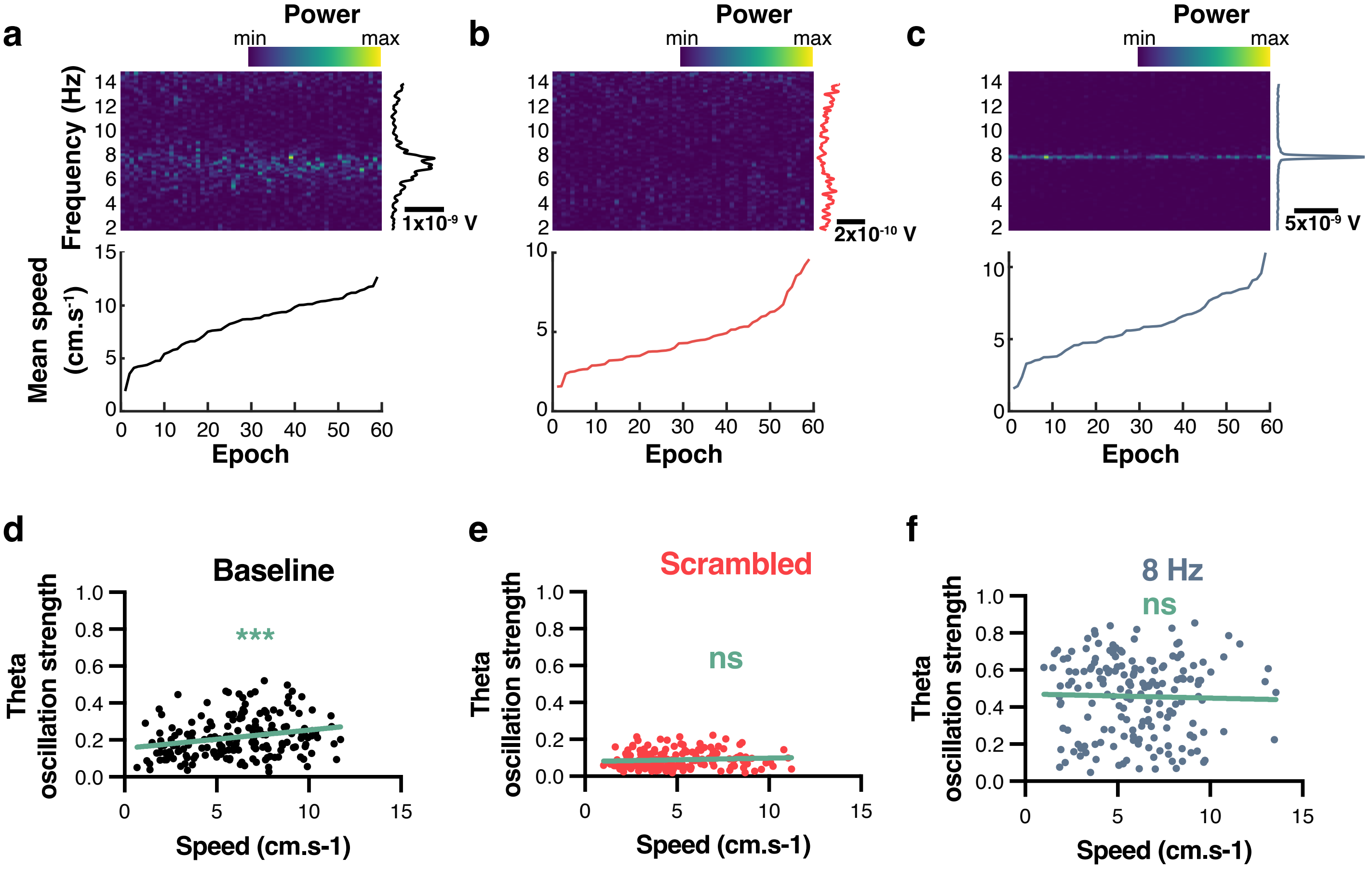

Supplementary figure 3. Optogenetic stimulations control theta irrespective of locomotor speed. a, baseline epochs containing natural theta. Top, spectrogram with x-axis sorted by average speed during recorded epoch (dark blue, low power; bright yellow, high power). Bottom, corresponding locomotor speed. **b**, same for epochs during scrambled stimulations. **c**, same for epochs during 8 Hz stimulations. **d**, theta oscillation strength against locomotor speed for baseline (Pearson $R^2 = 0.059$, $p = 0.001$; n = 179 independent epochs). **e**, theta oscillation strength against locomotor speed for scrambled stimulations (Pearson $R^2 = 0.008$, $p = 0.223$; n = 179 independent epochs). **f**, theta oscillation strength against locomotor speed for 8 Hz stimulations (Pearson $R^2 = 0.0008$, $p = 0.714$; n = 177 independent epochs).

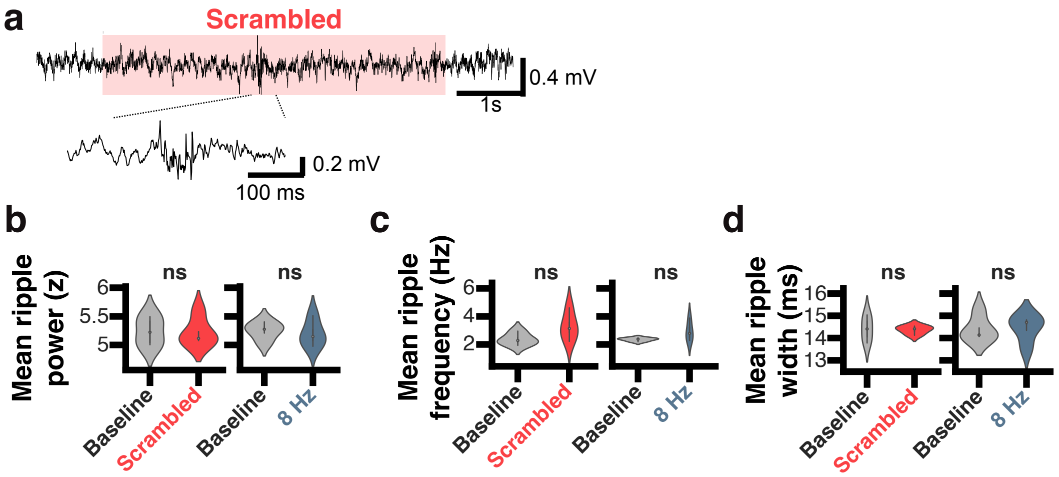

Supplementary figure 4. Characteristics of sharp wave ripples are not affected by optogenetic stimulations. a, example unfiltered trace of a ripple event during a scrambled optogenetic stimulation epoch. **b**, mean z-scored ripple power during baseline and either scrambled (red; unpaired, two-tailed t-test, $t_6 = 0.076$, $p = 0.941$) or 8 Hz (blue; unpaired, two-tailed t-test, $t_6 = 0.378$, $p = 0.718$) stimulations (N = 4 mice). **c**, same for mean ripple frequency of occurrence for scrambled (unpaired, two-tailed t-test, $t_6 = -1.688$, $p = 0.142$) and 8 Hz (unpaired, two-tailed t-test, $t_6 = -1.643$, $p = 0.151$) stimulations (N = 4 mice). **d**, mean ripple width during scrambled (unpaired, two-tailed t-test, $t_6 = 0.124$, $p = 0.905$) or 8 Hz (unpaired, two-tailed t-test, $t_6 = -0.138$, $p = 0.894$) stimulations (N = 4 mice).

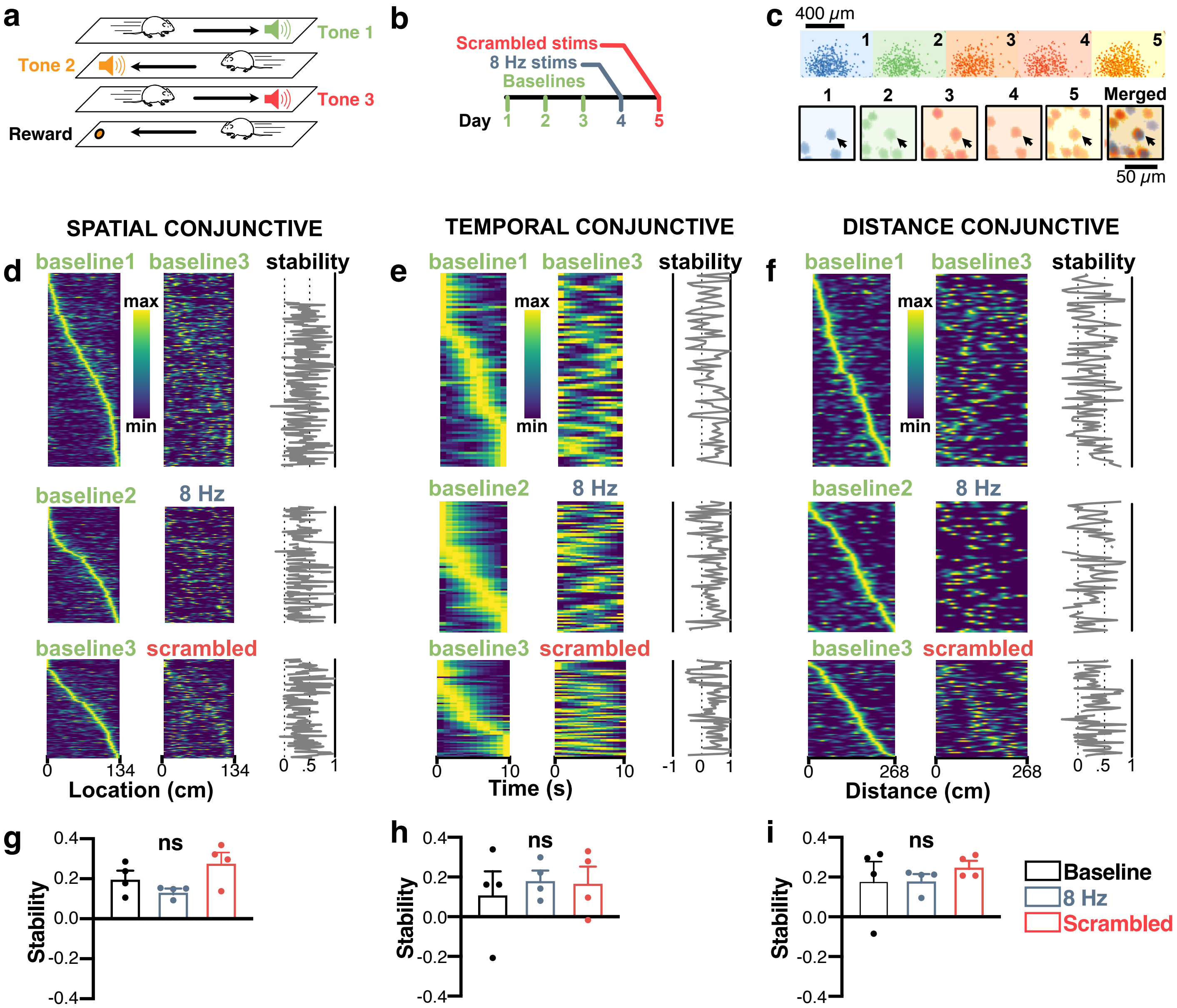

Supplementary figure 5: MS optogenetic stimulation does not disrupt the stability of spatiotemporal codes in conjunctive cells. a, mice were recorded on the 3-tone linear track to identify time-, place- and distance-modulated cells. **b**, experimental timeline. **c**, spatial footprints of neurons recorded over days and comparison scheme used to assess stability (top). Example neuron (black arrow) tracked over the 5 experimental days (bottom). **d**, sorted spatial tuning curves for identified conjunctive, spatially-modulated cells across day pairs (dark blue, low power; bright yellow, high power). The first day is used as a baseline and mice undergo 8 Hz, scrambled, or no stimulations (control treatment) during the other day. Stability is computed as the pairwise correlation of fields between the two-day pairs. **e**, same for conjunctive, time-modulated cells. **f**, same for conjunctive, distance-modulated cells. **g**, corresponding average stability for conjunctive, spatially-modulated cells (1ANOVA, $F_2 = 3.731$, $p = 0.0661$; N = 4 independent mice). **h**, same for conjunctive, time-modulated cells (1ANOVA, $F_2 = 1.993$, $p = 0.8228$; N = 4 independent mice). **i**, same for conjunctive, distance-modulated cells (1ANOVA, $F_2 = 0.469$, $p = 0.6400$; N = 4 independent mice). All bar plots represent mean ± SEM of at least two independent experiments.

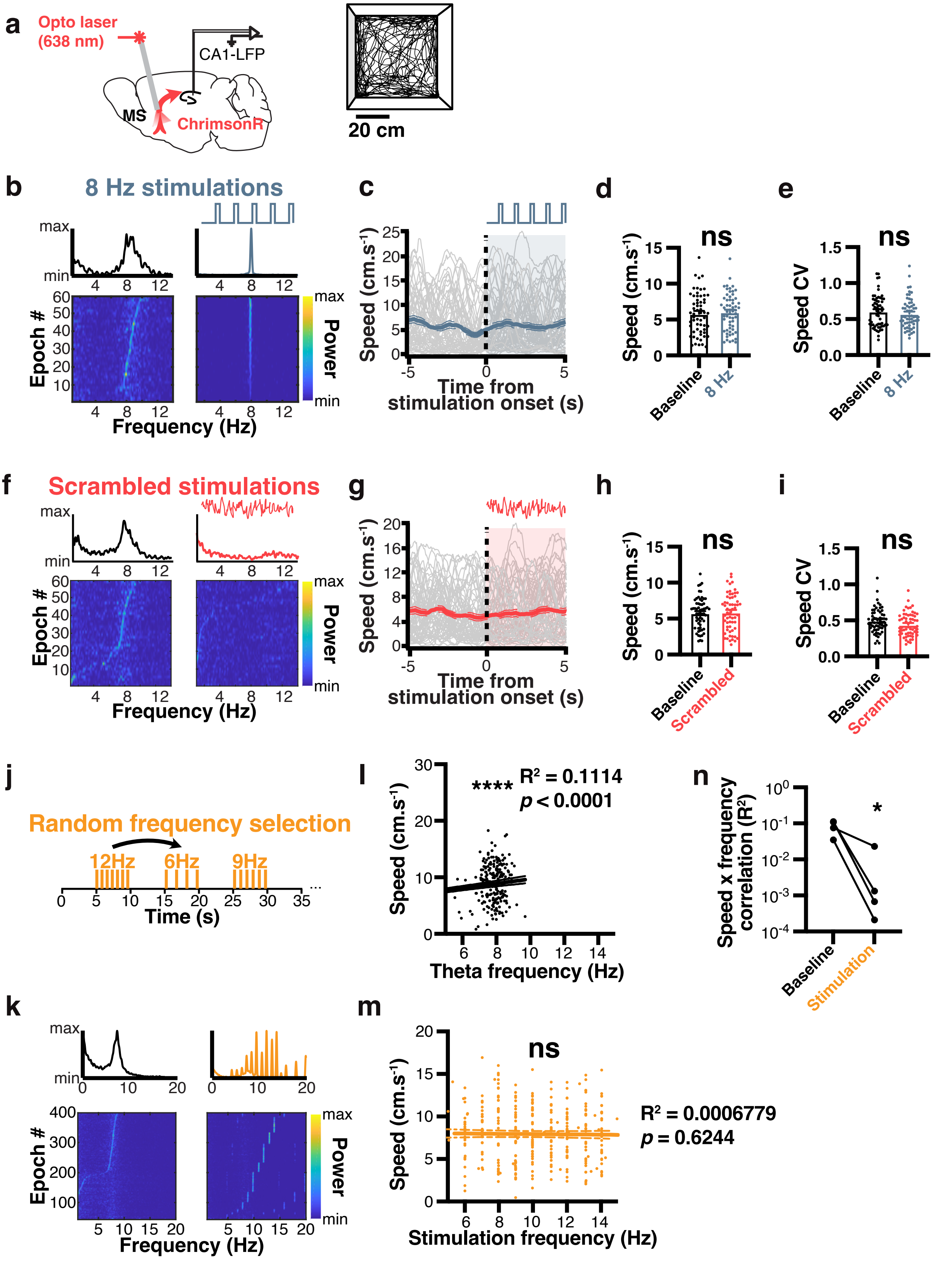
a
Opto laser (638 nm)
CA1-LFP
MS
ChrimsonR
20 cm
b
8 Hz stimulations
max
min
Epoch #
Power
Frequency (Hz)
c
Speed (cm.s-1)
Time from stimulation onset (s)
d
ns
Speed (cm.s-1)
Baseline
8 Hz
e
ns
Speed CV
Baseline
8 Hz
f
Scrambled stimulations
g
h
ns
Baseline
Scrambled
i
ns
j
Random frequency selection
12Hz
6Hz
9Hz
Time (s)
k
l
****
R2 = 0.1114
p < 0.0001
Theta frequency (Hz)
m
ns
R2 = 0.0006779
p = 0.6244
Stimulation frequency (Hz)
n
Speed x frequency correlation (R2)
*
Baseline
Stimulation

Supplementary figure 6. MS optogenetic stimulation does not control locomotor speed. a, mice were implanted with fiber optics in the MS after transfecting ChrimsonR, implanted with an electrode in CA1 (left), and freely explored an open field (right). **b**, average Fourier power spectra for 60 x 5s stimulation epochs (top), and sorted power spectra for each stimulation epoch (bottom; dark blue, low power; bright yellow, high power) for baseline (left, in black), and 8 Hz (right, in blue) stimulations in one example mouse. **c**, peristimulus time plot of locomotor speed before and during 8 Hz MS optogenetic stimulation. Average speed in blue (line thickness indicated SEM). **d**, average locomotor speed before and during 8 Hz MS optogenetic stimulation (unpaired, two-tailed t-test, $t_{116} = 0.4140$, $p = 0.6796$; n = 59 independent epochs). **e**, locomotor speed coefficient of variation (CV) before and during stimulation (unpaired, two-tailed t-test, $t_{116} = 0.8296$, $p = 0.4095$; n = 59 independent epochs). **f**, average Fourier power spectra 5s stimulation epochs (top), and sorted power spectra for each stimulation epoch (bottom; dark blue, low power; bright yellow, high power) for baseline (left, in black), and scrambled (right, in red) stimulations in one example mouse. **g**, peristimulus time plot of locomotor speed before and during scrambled MS optogenetic stimulation. Average speed in red (line thickness indicated SEM). **h**, average locomotor speed before and during scrambled MS optogenetic stimulation (unpaired, two-tailed t-test, $t_{118} = 0.2268$, $p = 0.8210$; n= 60 stimulation epochs). **i**, locomotor speed coefficient of variation (CV) before and during stimulation (unpaired, two-tailed t-test, $t_{118} = 1.838$, $p = 0.686$; n = 60 stimulation epochs). **j**, random frequency generator used to pace theta oscillations at varying frequencies (see Methods). **k**, average Fourier power spectra for 400 x 5s stimulation epochs (top), and sorted power spectra for each stimulation epoch (bottom; dark blue, low power; bright yellow, high power) for baseline (left, in black), and randomly selected (right, in orange) stimulations in one example mouse. **l**, relationship between locomotor speed and natural (unstimulated) theta frequency. **m**, same but during pacing of theta oscillations at varying stimulation frequencies. **n**, correlation between speed and natural (black) or controlled (orange) theta oscillations (paired t-test, $t_3 = 3.922$, $p = 0.0295$; N = 4 mice). All bar plots represent mean ± SEM of at least two independent experiments. Line plots and associates bands in **l** and **m** represent mean ± 95% confidence interval.

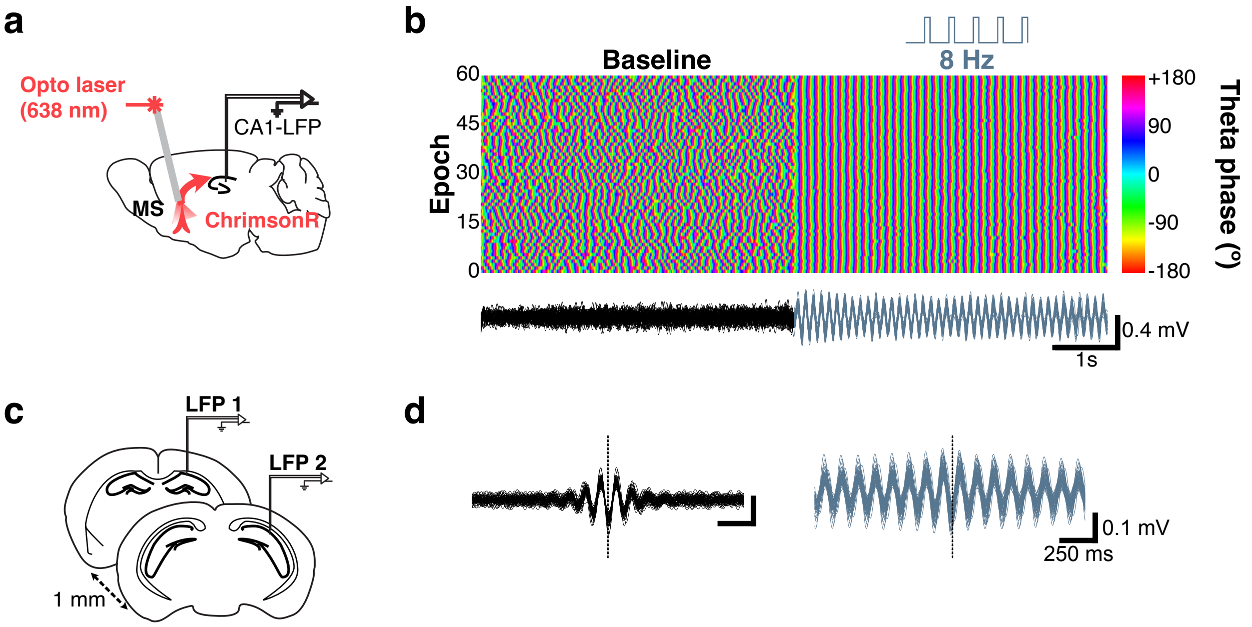

Supplementary fig. 7. 8 Hz optogenetic stimulations lead to non-physiological theta synchrony. a, mice were injected with ChrimsonR and implanted with a fiber optic in the MS. **b**, theta phase over time before and during optogenetic stimulation for (each horizontal line represents one of 60 epochs). Red, theta peak; cyan, theta trough. Bottom, corresponding traces. **c**, mice were implanted with two electrodes in dorsal CA1, 1 mm in the septotemporal axis. **d**, cross-correlation between the two recording sites (LFP1 and LFP2 in **c**) before (black traces) or during 8 Hz optogenetic stimulations.
