# Supplementary material for: Optogenetic frequency scrambling of hippocampal theta oscillations dissociates working memory retrieval from hippocampal spatiotemporal codes
Source: Nat Commun. 2023 Jan 25;14:410. doi: 10.1038/s41467-023-35825-5 (PMC9877037; doi:10.1038/s41467-023-35825-5)
Supplement: Supplementary file 3 — Reporting Summary [file 41467_2023_35825_MOESM3_ESM.pdf]

nature portfolio

Corresponding author(s): Sylvain Williams
Last updated by author(s): Dec 6, 2022

Reporting Summary

Nature Portfolio wishes to improve the reproducibility of the work that we publish. This form provides structure for consistency and transparency in reporting. For further information on Nature Portfolio policies, see our Editorial Policies and the Editorial Policy Checklist.

Statistics

For all statistical analyses, confirm that the following items are present in the figure legend, table legend, main text, or Methods section.

n/a	Confirmed	
☐	☒	The exact sample size (*n*) for each experimental group/condition, given as a discrete number and unit of measurement
☐	☒	A statement on whether measurements were taken from distinct samples or whether the same sample was measured repeatedly
☐	☒	The statistical test(s) used AND whether they are one- or two-sided<br>*Only common tests should be described solely by name; describe more complex techniques in the Methods section.*
☐	☒	A description of all covariates tested
☐	☒	A description of any assumptions or corrections, such as tests of normality and adjustment for multiple comparisons
☐	☒	A full description of the statistical parameters including central tendency (e.g. means) or other basic estimates (e.g. regression coefficient) AND variation (e.g. standard deviation) or associated estimates of uncertainty (e.g. confidence intervals)
☐	☒	For null hypothesis testing, the test statistic (e.g. *F*, *t*, *r*) with confidence intervals, effect sizes, degrees of freedom and *P* value noted<br>*Give P values as exact values whenever suitable.*
☐	☒	For Bayesian analysis, information on the choice of priors and Markov chain Monte Carlo settings
☒	☐	For hierarchical and complex designs, identification of the appropriate level for tests and full reporting of outcomes
☐	☒	Estimates of effect sizes (e.g. Cohen's *d*, Pearson's *r*), indicating how they were calculated

Our web collection on statistics for biologists contains articles on many of the points above.

Software and code

Policy information about availability of computer code

Data collection	Electrophysiological data was acquired using CHeetah 6.4.2 (Neuralynx). Calcium imaging data was acquired using open source Miniscope V3 DAQ software
Data analysis	Data analysis was performed using Mathworks MATLAB 2020a & Python 3.8. Statistical analyses were done with Graphpad Prism and Python module pingouin 0.5.1. All custom code is maintained on GitHub (https://github.com/), and publicly available: etterguillaume/etter_et_al_2022 (v1.0, cited in text); etterguillaume/MiniscopeAnalysis (v1.0); flatironinstitute/NoRMCorre (v0.1.1); zhoupc/CNMF_E (v1.1.2); zivlab/CellReg (v1.5.3)

For manuscripts utilizing custom algorithms or software that are central to the research but not yet described in published literature, software must be made available to editors and reviewers. We strongly encourage code deposition in a community repository (e.g. GitHub). See the Nature Portfolio guidelines for submitting code & software for further information.

Data

Policy information about availability of data

All manuscripts must include a data availability statement. This statement should provide the following information, where applicable:

- Accession codes, unique identifiers, or web links for publicly available datasets
- A description of any restrictions on data availability
- For clinical datasets or third party data, please ensure that the statement adheres to our policy

The processed dataset generated in this study is publicly available at https://osf.io/78wez/ or via request to the corresponding authors. Source data for all main text and Supplementary Figures can be found in the Source Data file Etter_NC2022_Source_Data.xlsx provided with this article.

Human research participants

Policy information about studies involving human research participants and Sex and Gender in Research.

Reporting on sex and gender	This study did not involve any human participant.
Population characteristics	See above
Recruitment	See above
Ethics oversight	See above

Note that full information on the approval of the study protocol must also be provided in the manuscript.

Field-specific reporting

Please select the one below that is the best fit for your research. If you are not sure, read the appropriate sections before making your selection.

☒ Life sciences ☐ Behavioural & social sciences ☐ Ecological, evolutionary & environmental sciences

For a reference copy of the document with all sections, see nature.com/documents/nr-reporting-summary-flat.pdf

Life sciences study design

All studies must disclose on these points even when the disclosure is negative.

Sample size	For calcium imaging and electrophysiological experiments, we ensured that sample size exceeded that of previous study for this type of analysis (Ziv et al., 2013). For the novel object task, we used typical sample size as previously described (Etter et al., 2019). For delayed non-match to sample, we used three times larger sample sizes than previously described for this task (Gemzik et al., 2021) and statistical tests leveraged intra-individual as well as inter-individual performances.
Data exclusions	In assessing performance in the tone-cued linear track, n=2 mice that did not perform 12 runs or more were excluded. In the novel object recognition task, n = 3 mice did not explore one of the two objects (<2s exploration) in either the sample or test phase, and were excluded. In the delayed non-match to sample task, n = 1 mouse never reached the training criterion (>80% correct choices) after 10 days of training, and was excluded.
Replication	All experiments were replicated at least twice, leading to the same results. Behavioral tests included at least three batches of mice tested at distinct dates, were not statistically different and were pooled. For calcium imaging, metrics such as portion of spatial/temporal cells were consistent across recording sessions and across mice (but were only pooled across mice). Optogenetic stimulations (combined with either electrophysiological or calcium imaging recordings) led to the same results on distinct testing dates and across mice.
Randomization	Mice were randomly attributed to a treatment group, regardless of their sex and cage of origin. In the delayed non-match to sample task, the treatment phase was randomly selected from day to day. In the novel place object recognition task, the location and identity of the displaced object was randomly selected for each mouse
Blinding	In behavioral experiments (DNMTS and NPOR), a dedicated experimenter performed the assays while being blind to the identity and treatment of tested groups. For calcium imaging and electrophysiological experiments, identification numbers where coded by another experimenter to prevent identification of subjects during analysis

Reporting for specific materials, systems and methods

We require information from authors about some types of materials, experimental systems and methods used in many studies. Here, indicate whether each material, system or method listed is relevant to your study. If you are not sure if a list item applies to your research, read the appropriate section before selecting a response.

Materials & experimental systems

n/a	Involved in the study
☐	☒ Antibodies
☒	☐ Eukaryotic cell lines
☒	☐ Palaeontology and archaeology
☐	☒ Animals and other organisms
☒	☐ Clinical data
☒	☐ Dual use research of concern

Methods

n/a	Involved in the study
☒	☐ ChIP-seq
☒	☐ Flow cytometry
☒	☐ MRI-based neuroimaging

Antibodies

Antibodies used	anti-Parvalbumin, monoclonal IgG1 clone PARV-19 produced in mouse (Sigma-Aldrich, catalog number: P3088)<br>anti-Choline acetyltransferase produced in goat (Millipore, AB144P)<br>got anti-mouse IgG1 coupled to Alexa 555 (Life Technologies, A21127)<br>donkey anti-goat coupled to Alexa 647 (Jackson Immunoresearch, 705-605-147)
Validation	Anti-parvalbumin was cross-validated by performing the immunostaining in a PVCre mouse crossed with a tdTomato-lox reporter mouse and validating labeling of tomato+ neurons in the medial septum. Further validation was previously performed: PMIDs: 31757962, 26050044.<br>Similarly, anti-ChAT primary antibody was cross-validated by performing the immunostaining in ChAT-cre mouse crossed with a tdTomato reporter mouse and validating labeling of tomato+ neurons in the diagonal band of Broca. Further validation was previously performed: 27617738.<br>Secondary antibodies were controlled for non-specific labeling by incubating secondary antibodies without initial primary antibody incubation. In these conditions, we observed no fluorescence signal, validating that secondary antibodies had to bind to their respective primary antibody to observe signal.

Animals and other research organisms

Policy information about studies involving animals; ARRIVE guidelines recommended for reporting animal research, and Sex and Gender in Research

Laboratory animals	8-16 weeks old male (n = 20) and female (n = 21) B6;129P2 parvalbumin-cre (Jackson Laboratory RRID:IMSR_JAX:017320) were used in this study
Wild animals	No wild animals were used in this study
Reporting on sex	Mice of both sexes were considered in this study. With no particular justification for focusing on one sex, we designed our study to include a balanced representation of both sexes, and results presented in this study apply to both sexes. Sex differences were not the focus of this study, sample size was determined to
Field-collected samples	No field-collected samples were used in this study
Ethics oversight	All procedures were approved by the McGill University Animal Care Committee and the Canadian Council on Animal Care (protocol 2015-7650).

Note that full information on the approval of the study protocol must also be provided in the manuscript.
